# Estimation and visualization of process states using latent variable models based on Gaussian process

**DOI:** 10.1002/ansa.202000122

**Published:** 2021-01-28

**Authors:** Hiromasa Kaneko

**Affiliations:** ^1^ Department of Applied Chemistry, School of Science and Technology Meiji University Kanagawa Japan

**Keywords:** dynamics, Gaussian process, latent variable, machine learning, process state estimation, visualization

## Abstract

The estimation and visualization of process states are important for process control in chemical and industrial plants. Since industrial processes are related to Gaussian distributions theoretically, this study focused on Gaussian process latent variable models. Process state estimation and visualization methods are proposed using two latent variables based on the Bayesian Gaussian process latent variable model (BGPLVM), infinite warped mixture model (iWMM), and Gaussian process dynamical models (GPDM). The Tennessee Eastman process dataset was analyzed and it was confirmed that the performance of estimating the process states was highest in the order of GPDM, iWMM, and BGPLVM. Moreover, time‐delayed process variables were added to the process variables to consider the process dynamics, which further improved the performance of estimating the process states. Particularly in the case of GPDM, only two latent variables could estimate the process states, with approximately 100% accuracy for four process states. Additionally, even 10 process states could be estimated with approximately 90% accuracy, and it was confirmed that the process state estimation and process state visualization could be achieved simultaneously.

## 1 INTRODUCTION

In chemical and industrial plants, process variables such as the temperature, pressure, flow rate, and liquid level are measured, and the measured time series data are used to control and manage the plants. For example, the data in the normal process state in a plant are used to define and model the data region of process variables representing normal process states. Process states are modes where plants are operated and controlled, and this model is used to detect the process faults in plants.^1^ Linear methods such as principal component analysis (PCA)^2^ and independent component analysis (ICA)^3^ are used for modeling. Dynamic PCA^4^ and dynamic ICA^5^ also exist, and are modeled by adding time‐delayed variables to consider the process dynamics. Nonlinear methods, such as the kernel PCA,^6^ k‐nearest neighbor algorithm,^7^ one‐class support vector machine (OCSVM),^8^ and self‐organizing map (SOM),^9^ can be used to consider the nonlinearity between process variables.

Once the process faults are detected, the process variables related to the detected faults should be diagnosed to clarify the cause of the faults. Fault diagnosis methods include a linear PCA‐based method^10^ and a nonlinear SOM‐based method.^9^ Additionally, there exist fault diagnosis methods based on process fault databases using dynamic simulators^11^ and R vine and Bayesian networks.^12^ Yin and Jiang reviewed recent advances in key performance indicator oriented prognosis and diagnosis,^13^ and developed a MATLAB toolbox, which is data‐based key performance indicator oriented fault detection toolbox (DB‐KIT) (https://www.mathworks.com/matlabcentral/fileexchange/65348-db-kit).

Soft sensors can be used to rapidly control the difficult‐to‐measure process variables *y* in a plant, such as the concentration and density. A soft sensor is a regression model constructed between *y* and the easy‐to‐measure process variables *x*, such as the temperature, pressure, flow rate, and liquid level. By inputting *x* values measured in real time into the model, the *y* values can be predicted and the predicted *y* values can be used for process control. Linear regression analysis methods include partial least‐squares regression,^14^ ridge regression, least absolute shrinkage and selection operator, and elastic nets,^15^ while nonlinear approaches include support vector regression,^16^ random forests (RFs),^17^ and deep neural networks.^18^ Adaptive soft sensors^19^ have been developed to adapt the soft sensors to the process state changes in a plant.

It is important to estimate the process states in plants for fault detection, fault diagnosis, and soft sensors. For example, in polymer production plants, polymers of multiple grades are produced in a single reactor, and the process states differ depending on the grades, even though each state is normal and stable. When detecting and diagnosing process faults, it is necessary to assess whether the current process state is normal or abnormal, even when there are several normal states. Additionally, it has been reported that the relationships between *x* and *y* change depending on the process states in soft sensor analyses, and a model constructed for each local process state can improve the predictive ability of a soft sensor compared with a single global model.^20^ Jiang et al. reviewed soft sensors for monitoring, control, and optimization of industrial processes.^21^

The results of fault detection, fault diagnosis, soft sensors, and process state estimation must be visualized such that they can be used by plant operators. Because process data are multidimensional, a dataset is mapped onto a two‐dimensional plane to check the process states by visual contact. By confirming the visualization result, we can check the current process state and the transition of process states. The objective of this study was to estimate the process states and visualize them.

Nikula et al. estimated process states in steam boiler using selected process variables to monitor the boiler performance.^22^ The process states should be estimated to design model predictive control.^23^ Moreover, the process transitions are estimated and the process states are identified using dynamic locus analysis.^24^ Time‐explicit Kiviat plots are used to detect process faults and visualize a dataset.^25^ The *t*‐distributed stochastic neighbor embedding technique is used to transform the process variables into a two‐dimensional space and visualize the process datasets.^26^

This study focused on Gaussian process latent variable models (GPLVMs)^27^ for process state estimation and visualization since industrial processes are related to Gaussian distributions theoretically. The GPLVMs are an unsupervised learning method based on the Gaussian process of transforming process data to latent variables *z* with consideration to the probability density of a dataset. Additionally, nonlinear transformations are possible using kernel functions. Furthermore, although the general prior distribution of *z* is assumed to be a standard normal distribution, the wide variety of *z* can be calculated by changing the prior distribution of *z*. For example, infinite warped mixture models (iWMM)^28^ assume a mixture of Gaussians for the prior distribution of *z*, and Gaussian process dynamical models (GPDM)^29^, ^30^ assume Markov processes for the prior distribution of *z*. The Bayesian GPLVM (BGPLVM),^31^ which uses the variational Bayesian method to train the posterior distribution and hyperparameters of a model, has also been proposed. The GPLVM has been applied to fault detection,^32^ and GPDM has been applied to adaptive soft sensors.^33^

In this study, the process state estimation and visualization are investigated using BGPLVM, iWMM, and GPDM. Additionally, time‐delayed process variables are added to consider the process dynamics. Additionally, the process states are estimated and visualized using a dataset of multiple process faults in the Tennessee Eastman process (TEP).^34^

## 2 METHOD

The methods utilized in this study are basically GPLVM, but iWMM and GPDM are derived from it, changing kernel functions. First, the theoretical aspects of the Gaussian process are explained, and then, GPLVM, iWMM, and GPDM are described.

### 2.1 Gaussian process

When the *k*th *x* is *x_k_*, and the weight of the *i*th *z* with respect to *x_k_* is *b_i_*, *x_k_* is assumed to be given by the linear combination of *z*. *b_i_* is normally distributed, and thus, there exist *m* Gaussian distributions (*m* is the number of z). For all *m* Gaussian distributions, the mean is 0 and the variance is *σ*_b_^2^. The covariance between *b_i_* and *b_j_* is 0. *x_k_* ^(^ *^i^* ^)^, which is *x_k_* in the *i*th sample, is given as follows:

(1) $$x_{k}^{\left(i\right)}=z^{\left(i\right)}b,$$

where **z** ^(^ *^i^* ^)^ is the vector of *z* in the *i*th sample and **b** is the weight vector.

For a Gaussian distribution of *x_k_* ^(^ *^i^* ^)^, the mean *μ_k,i_* is given as follows:

(2) $$\mu_{k,i}=E\left[x_{k}^{\left(i\right)}\right]=E\left[z^{\left(i\right)}b\right].$$

Equation (2) is transformed as follows:

(3) $$\mu_{i}=x^{\left(i\right)}E\left[b\right]=0.$$

The covariance *σ*_x_ *_k_*_,_ *_i_*_,_ *_j_* ^2^ between *x_k_* ^(^ *^i^* ^)^ and *x_k_* ^(^ *^j^* ^)^ is given as follows:

(4) $${\sigma_{k,i,j}}^{2}=\mathrm{cov}\left[x_{k}^{\left(i\right)},x_{k}^{\left(j\right)}\right]=\mathrm{cov}\left[z^{\left(i\right)}b,z^{\left(j\right)}b\right].$$

Equation (4) is transformed as follows:

(5) $${\sigma_{k,i,j}}^{2}=E\left[z^{\left(i\right)}b{\left(z^{\left(j\right)}b\right)}^{T}\right]=E\left[z^{\left(i\right)}bb^{T}{z^{\left(j\right)}}^{T}\right]=z^{\left(i\right)}E\left[bb^{T}\right]{z^{\left(j\right)}}^{T}={\sigma_{b}}^{2}z^{\left(i\right)}{z^{\left(j\right)}}^{T}.$$

*x* often contains measurement errors. It is assumed that measurement errors follow the Gaussian distribution with the mean of 0 and the variance of *σ*_e_^2^. When the Gaussian distribution of measurement errors in the *i*th sample is *e* ^(^ *^i^* ^)^, the measured *x_k_* ^(^ *^i^* ^)^, which is *x*_obs,_ *_k_* ^(^ *^i^* ^)^, is given as follows:

(6) $${x_{\mathrm{obs},k}}^{\left(i\right)}=x_{k}^{\left(i\right)}+e^{\left(i\right)}$$

From Equation (3), the mean of *x*_obs,_ *_k_* ^(^ *^i^* ^)^ is 0, and from Equation (5), the covariance between *x*_obs,_ *_k_* ^(^ *^i^* ^)^ and *x*_obs,_ *_k_* ^(^ *^i^* ^)^ is given as follows:

(7) $$\sigma^{2}z^{\left(i\right)}z^{\left(j\right)T}+\delta_{i,j}{\sigma_{e}}^{2},$$

where *δ_i_*_,_ *_j_* is the Kronecker delta.

The relationships between samples in *z* are represented by the relationships in *x*, which is called the Gaussian process.

### 2.2 Gaussian process latent variable model

In GPLVMs, samples with similar *z* values are also considered to have similar *x* values, and it is assumed that the relationship of the *x* values among the samples can be calculated from the relationship of the *z* values. Assuming that each *x* is generated by Gaussian process regression^16^ from *z*, the mean of *x* in the *i*th sample is zero and the covariance of *x* between the *i*th sample and the *j*th sample (or the variance when *j *= *i*) is calculated from the *z* values of the *i*th sample and *j*th sample, which is expressed as follows:

(8) $$K\left(z^{\left(i\right)},\;z^{\left(j\right)}\right)=\sigma^{2}z^{\left(i\right)}z^{\left(j\right)T}+\delta_{i,j}{\sigma_{e}}^{2},$$

where **z** ^(^ *^i^* ^)^ ϵ *R^m^* denotes the latent variables of the *i*th sample (*m* is the number of the latent variables), *σ* ^2^ denotes the variance of the regression coefficients, *δ_i_*_,_ *_j_* is the Kronecker delta, and *σ*_e_^2^ is the variance of noise in *x*. To consider the nonlinearity of *x* and *z*, Equation (8) is calculated using a kernel function. In this study, the following kernel function was used:

(9) $$K\left(z^{\left(i\right)},\;z^{\left(j\right)}\right)=\theta_{0}\exp\left\{-\frac{\theta_{1}}{2}{\left\|z^{\left(i\right)}-z^{\left(j\right)}\right\|}^{2}\right\}+\theta_{2}+\theta_{3}\sum_{k=1}^{m}{z_{k}}^{\left(i\right)}{z_{k}}^{\left(j\right)},$$

where *θ*_0_, *θ*_1_, *θ*_2_, and *θ*_3_ are the hyperparameters.

The probability for the occurrence of *x* values in samples 1, 2, …, *i*, …, and *n* given *z* is calculated as follows:

(10) $$\begin{array}{cc} p\left(x|z\right) & =\prod_{i=1}^{n}p\left(x^{\left(i\right)}|z\right)\\ & =\prod_{i=1}^{n}N\left(x^{\left(i\right)}|0,\;\;K\right) \end{array},$$

where **x** ^(^ *^i^* ^)^ ϵ *R^p^* is a vector of *x* variables in the *i*th sample (*p* is the number of *x* variables), and **K** ϵ *R^m×m^* is the gram matrix whose elements are values of the kernel function. As a prior distribution of *z*, it is assumed that each **z** ^(^ *^i^* ^)^ follows a standard Gaussian distribution in the *m* dimension as follows:

(11) $$p\left(z\right)=\prod_{i=1}^{n}p\left(z^{\left(i\right)}\right),$$

(12) $$p\left(z^{\left(i\right)}\right)=N\left(0,\;\;E\right),$$

where **E** ϵ *R^m×m^* is a unit matrix. In GPLVM, *z* is calculated to maximize the following joint probability of *x* and *z*:

(13) $$\begin{array}{cc} p\left(x,z\right) & =p\left(x|z\right)p\left(z\right)\\ & =\prod_{i=1}^{n}N\left(x^{\left(i\right)}|0,\;\;K\right)\times \prod_{i=1}^{n}p\left(z^{\left(i\right)}\right) \end{array}.$$

The BGPLVM uses a variational Bayesian method to train the posterior distribution of *z* and the hyperparameters.

The method of calculating *z* with BGPLVM by adding time‐delayed process variables to *x* is called dynamic BGPLVM (DBPLVM). The GPy Python library (https://sheffieldml.github.io/GPy/) was used for BGPLVM.

### 2.3 Infinite warped mixture model

In iWMM, it is assumed that the prior distribution of *z* follows a Gaussian mixture model wherein each **z** ^(^ *^i^* ^)^ has *a* components. Instead of Equation (12), the following equation is used:

(7) $$p\left(z^{\left(i\right)}\right)=\sum_{k=1}^{a}\pi_{k}N\left(z^{\left(i\right)}|\mu_{k},\Sigma_{k}\right),$$

where *a* is the number of Gaussians, and **μ** *_k_* and **Σ** *_k_* are the mean vector and variance‐covariance matrix of the *k*th Gaussian, respectively; *π_k_* is the weight of the *k*th Gaussian provided according to the following conditions:

(8) $$0\leq \pi_{k}\leq 1,\;\;\;\;\sum_{k=1}^{n}\pi_{k}=1.$$

The method for calculating *z* with iWMM by adding time‐delayed process variables to *x* is called dynamic iWMM (DiWMM). Warped‐mixtures (https://github.com/duvenaud/warped-mixtures/) were used as a MATLAB library of iWMM.

### 2.4 Gaussian process dynamical model

In GPDM, *z* is considered to be time series data evolving through time, and *x* is considered to evolve through time according to a nonlinear Markov process *p*(**z** ^(^ *^i^* ^)^|**z** ^(^ *^i^* ^−1)^). Instead of Equation (11), the following equation is used:

(9) $$p\left(z\right)=\prod_{k=2}^{n}p\left(z^{\left(i\right)}|z^{\left(i-1\right)}\right),$$

where *p*(**z** ^(^ *^i^* ^)^|**z** ^(^ *^i^* ^−1)^) is represented by the kernel function.

The method for calculating *z* with GPDM by adding time‐delayed process variables to *x* is called dynamic GPDM (DGPDM). The GPDM code (http://www.dgp.toronto.edu/~jmwang/gpdm/) was used as a MATLAB library for GPDM.

### 2.5 Modeling using the GPLVM methods

When the proposed method is applied to process data, a dataset of *x* is prepared and BGPLVM, iWMM, and GPDM are executed. When the dynamics of processes is considered, time‐delayed variable is added to *x* in the preparation of a dataset.

As a limitation of BGPLVM, iWMM, and GPDM, *z* can be calculated from *x* for a given dataset; however, the model cannot be used to predict *z* for a new sample. When it is necessary to calculate *z* in a new sample, a new sample is added to the existing dataset and BGPLVM, iWMM, and GPDM are executed again.

## 3 RESULTS AND DISCUSSION

To verify the effectiveness of the Gaussian process based latent variable models, the TEP dataset was analyzed. The Eastman Chemical Company developed the TEP data to mimic an actual industrial process, and this dataset has been used to compare the performance of process control and monitoring methods. Notably, TEP comprises the data for the reactor, condenser, compressor, separator, and stripper, with a total of eight components (A through H). The liquid products, G and H, and the by‐product F, are generated from the gaseous reactants A, C, D, and E through chemical reactions. Additionally, 22 process variables and 19 composition measurements are used as input variables.

There are 21 preprogrammed process faults in the TEP database. This study focused on nine process faults or variations: the A/C feed ratio and B percentage (fault 1); percentage of B and A/C ratio (fault 2); reactor cooling water inlet temperature (fault 4); feed loss (fault 6); C header pressure loss‐reduced availability (fault 7); A, B, and C feed composition (fault 8); condenser cooling water inlet temperature (fault 12); reaction kinetics (fault 13); reactor cooling water valve (fault 14). The data for each process fault comprise 360 (1080 min) observations, including the initial 160 (480 min) normal state data points. These data are the same as those previously reported in the literature. In this study, four dataset types, including multiple process states, were considered as follows:

- four process states: normal state, fault 2, fault 6, fault 7;
- six process states: normal state, fault 1, fault 2, fault 6, fault 7, fault 8;
- eight process states: normal state, fault 1, fault 2, fault 6, fault 7, fault 8, fault 12, fault 13;
- 10 process states: normal state, fault 1, fault 2, fault 4, fault 6, fault 7, fault 8, fault 12, fault 13, fault 14.

The following models were used to extract the latent variables: PCA, dynamic PCA (DPCA), BGPLVM, DBGPLVM, iWMM, DiWMM, GPDM, and DGPDM. Process variables with a delay of 1 and 2^4^ were included for DPCA, DBGGPLVM, DiWMM, and DGPDM. Although GPDM allows the consideration of process dynamics in the calculation of latent variables, DGPDM was also considered for consistency with the other methods.

Only two latent variables should be used to visualize the process states. Therefore, to verify whether the two latent variables calculated with each method can accurately estimate the process states, each process state in each dataset was classified using only two latent variables and a classification method. For each process state in each dataset, the training data were 360 samples in a batch, and the test data were 360 samples in another batch. The RF models were constructed using the training data, and the accuracy rate in the prediction of the test data is presented in Table 1. In approximately all results presented in Table 1, the addition of time‐delayed process variables improved the accuracy rate. Thus, it was confirmed that the process states can be more appropriately estimated by considering the dynamics in the process of a plant. In all datasets, the accuracy rate of the latent variable model based on the Gaussian process was higher than that of PCA and DPCA, respectively. Furthermore, among the latent variable models based on the Gaussian process, BGPLVM, iWMM, and GPDM tended to be highest in the order of BGPLVM, iWMM, and GPDM. While BGPLVM assumes a single multidimensional Gaussian distribution in the space of the latent variables, iWMM assumes a mixture of multidimensional Gaussian distributions. The mixture of Gaussians allows us to appropriately consider the process states. By properly considering the process dynamics using GPDM, the estimation performance of the process states was further improved. Specifically, DGPDM estimated the process states with approximately 100% accuracy for the four process states, and with approximately 90% accuracy even when the number of process states was 10.

**TABLE 1:** Accuracy rate in test data of each number of process states for RF using only two latent variables calculated with each method

	4	6	8	10
PCA	0.883	0.789	0.718	0.627
DPCA	0.926	0.819	0.777	0.663
BGPLM	0.957	0.902	0.788	0.701
DBGPLM	0.969	0.901	0.864	0.720
iWMM	0.951	0.922	0.832	0.795
DiWMM	0.963	0.929	0.903	0.826
GPDM	0.985	0.978	0.935	0.866
DGPDM	0.994	0.972	0.953	0.896

**FIGURE 1:**
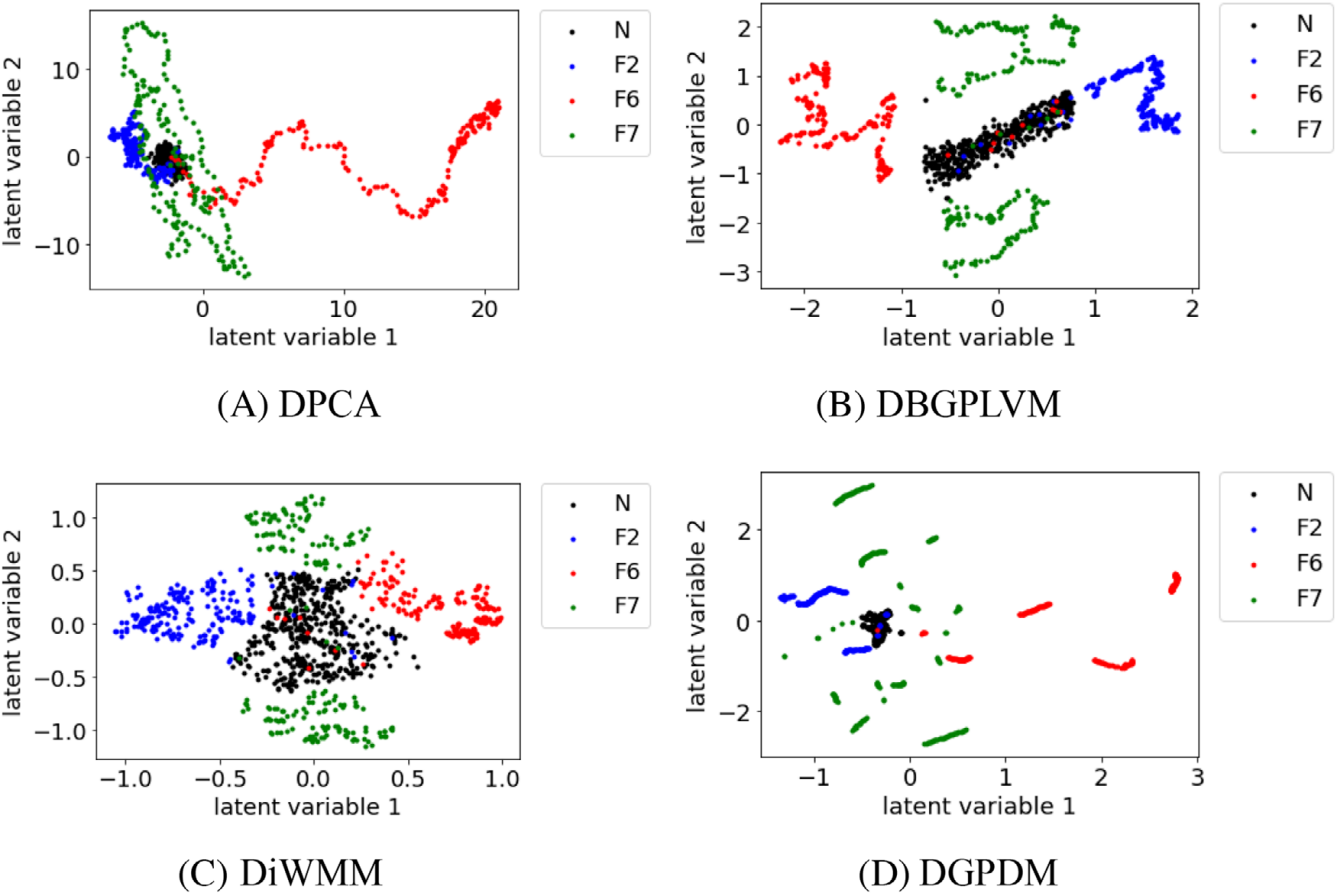
Data visualization with DPCA, DBGPLVM, DiWMM, and DGPDM when there are four process states. N and F in legends mean normal state and fault, respectively

**FIGURE 2:**
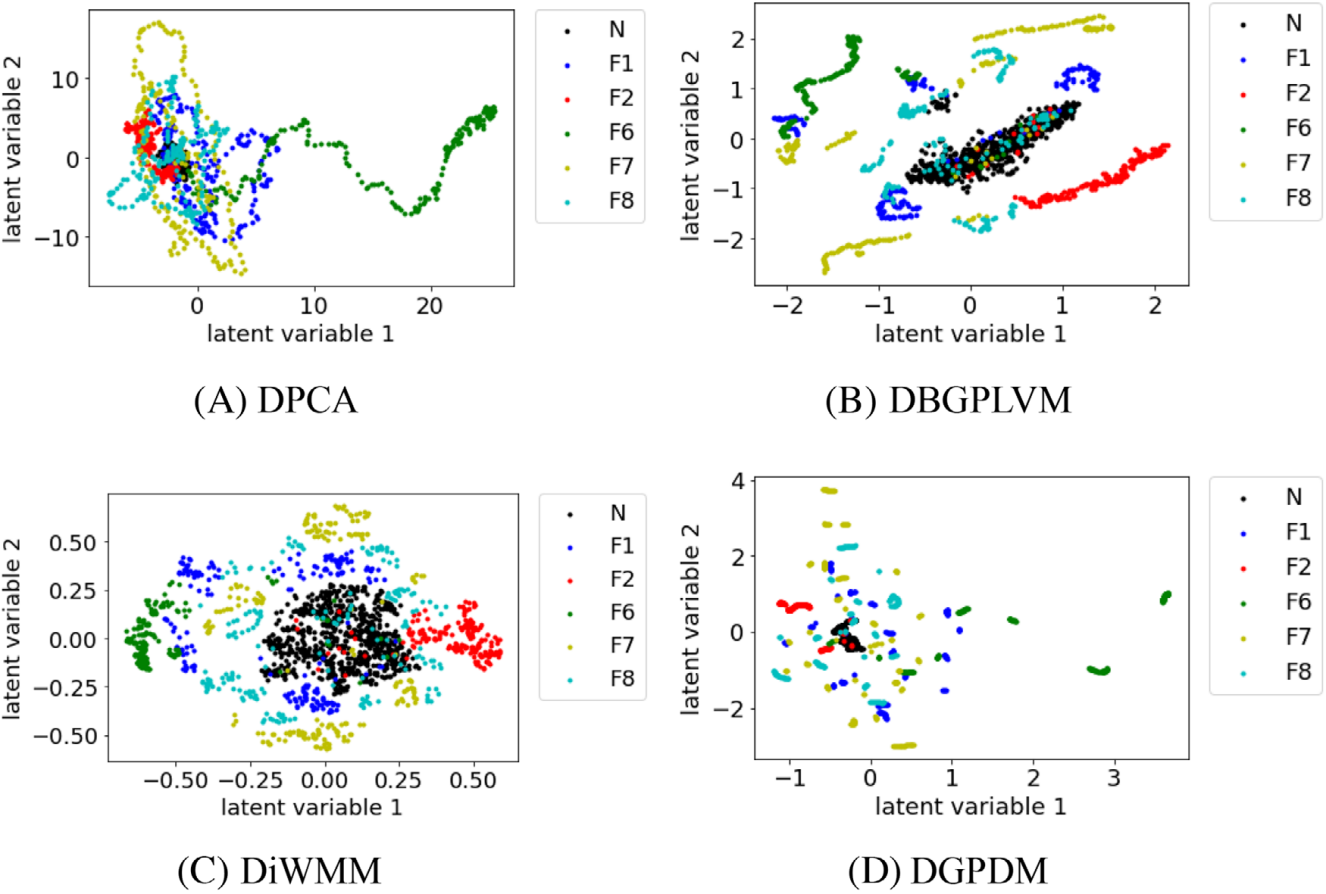
Data visualization with DPCA, DBGPLVM, DiWMM, and DGPDM when there are six process states. N and F in legends mean normal state and fault, respectively

**FIGURE 3:**
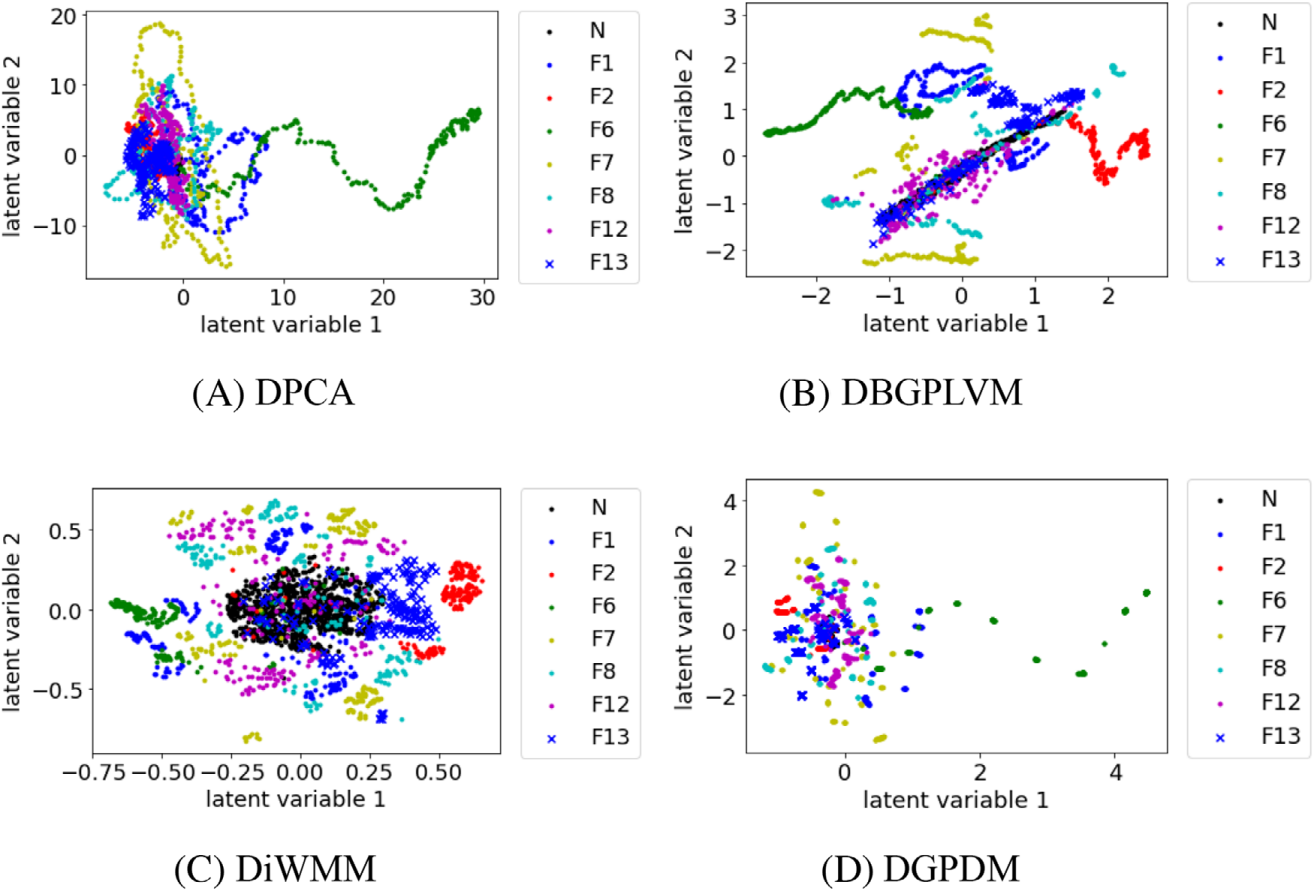
Data visualization with DPCA, DBGPLVM, DiWMM, and DGPDM when there are eight process states. N and F in legends mean normal state and fault, respectively

**FIGURE 4:**
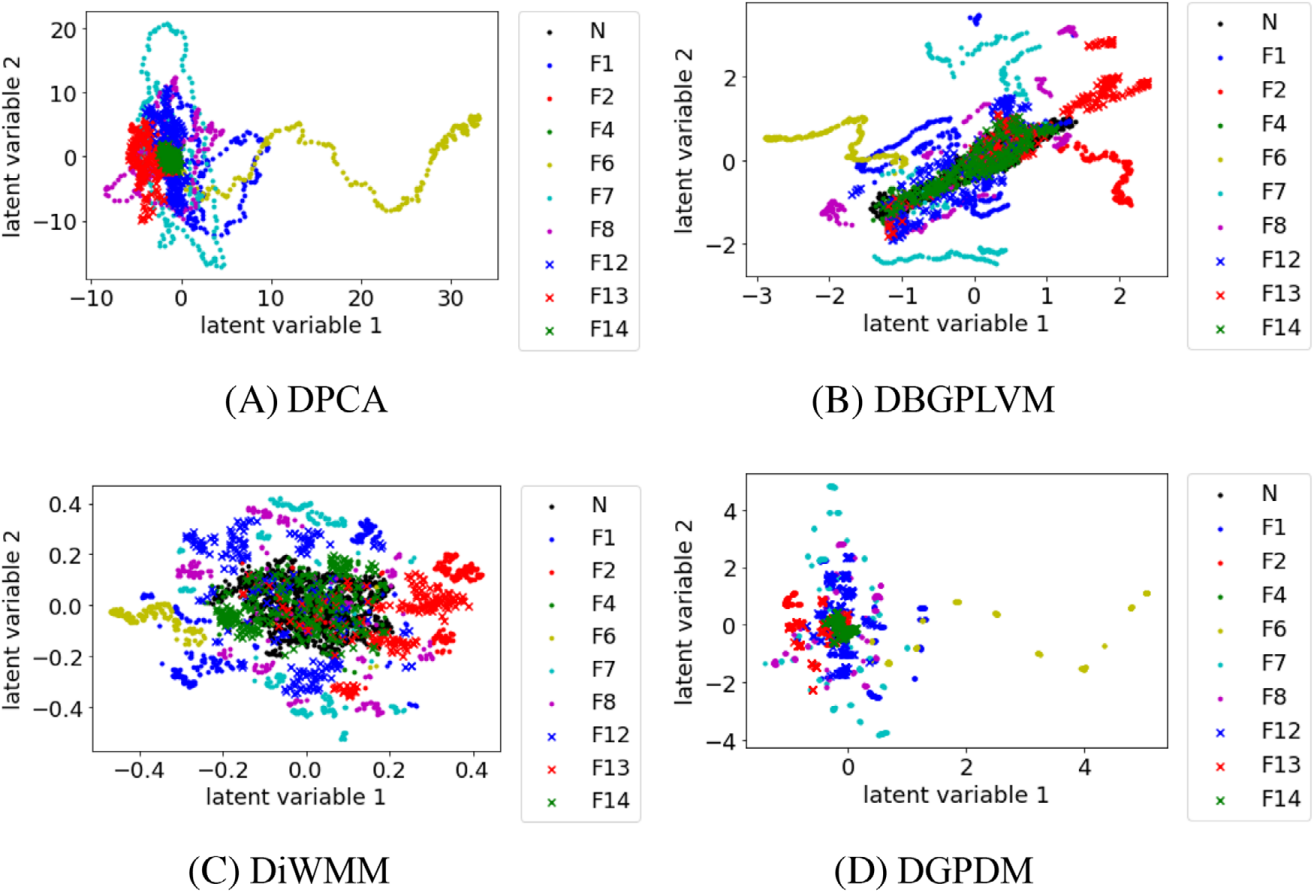
Data visualization with DPCA, DBGPLVM, DiWMM, and DGPDM when there are 10 process states. N and F in legends mean normal state and fault, respectively

Figures 1, 2, 3, 4 show the scatter plots of the latent variables in DPCA, DBGPLVM, DiWMM, and DGPDM for each dataset. The different colors of the points indicate different process states. From Figure 1, it can be seen that, in DPCA, many of the yellow points overlapped with the points indicated in other colors, which suggests that the dataset visualization failed. In DBGPLVM, DiWMM, and DGPDM, the samples of each process state were located in different regions, which suggests that the dataset was properly visualized.

Figures 2, 3, 4 show that, when the number of process states increased to 6, 8, and 10, there existed substantial overlap among the samples in the data distribution between each process state in DPCA. In DBGPLVM, there existed substantial overlap in the data distribution per process state, particularly when the number of process states was eight or more. In DiWMM, the samples of each abnormal process state were located around the data distribution of the normal process state, which indicates that the process states were properly visualized. In DGPDM, there existed regions of localized sample clumping in the same process state. Thus, it was confirmed that DiWMM and DGPDM could properly visualize the process states, even when there existed many process states among the latent variable models based on the Gaussian process.

## 4 CONCLUSION

To achieve the estimation and visualization of process states in chemical and industrial plants, this study investigated the latent variable models based on the Gaussian process, namely, BGPLVM, iWMM, and GPDM. As validated using the TEP dataset, GPDM, iWMM, and BGPLVM can achieve better performance in estimating the process states with only two latent variables in the order of GPDM, iWMM, and BGPLVM. With the addition of time‐delayed process variables, DBGPLVM, DiWMM, and DGPDM were also investigated to consider the process dynamics. The performance of estimating the process states improved compared with the performance before adding the time‐delayed process variables. Particularly, when using DGPDM, it was confirmed that only two latent variables were used to estimate the process states and approximately 100% accuracy was achieved with four process states. Even with 10 process states, the process states could be estimated with approximately 90% accuracy, and it was confirmed that both the estimation and visualization of the process states could be achieved simultaneously.

It is expected that process control can become more efficient by estimating and visualizing process states using latent variable models based on the Gaussian process.
